# An open-access future for *Journal of Synchrotron Radiation* – Editorial from the Main Editors and IUCr Journals Editor-in-Chief

**DOI:** 10.1107/S1600577521007086

**Published:** 2021-08-16

**Authors:** Kristina Kvashnina, Yoshiyuki Amemiya, Dibyendu Bhattacharyya, Ingolf Lindau, Andrew J. Allen

**Affiliations:** a Rossendorf Beamline (BM20), The European Synchrotron (ESRF), 71 Avenue des Martyrs, 38000 Grenoble, France; b Japan Synchrotron Radiation Research Institute (JASRI), 1-1-1 Kouto, Sayo-gun, Hyogo 679-5198, Japan; c Atomic and Molecular Physics Division, Bhabha Atomic Research Centre, Mumbai 400094, India; d SLAC/Stanford University, 2575 Sand Hill Road, MS69, Menlo Park, CA 94025, USA; e Materials Measurement Science Division, Stop 8520, National Institute of Standards and Technology, 100 Bureau Drive, Gaithersburg, MD 20899-8523, USA

**Keywords:** *Journal of Synchrotron Radiation*, open access, Editorial

## Abstract

Discussing *JSR*’s forthcoming transition to open access.

The entire *Journal of Synchrotron Radiation* (*JSR*) editorial team would like to take this opportunity to inform all our readers, authors and supporters about the coming transition to open access. All papers submitted to *JSR* after 1 October 2021 will be for open-access publication. By taking this step, *JSR* is supporting a journey towards open science in general.

*JSR* was founded in 1994 (Hasnain, Helliwell & Kamitsubo, 1994 ▸) with the aim of providing comprehensive coverage of the entire field of synchrotron radiation. Almost immediately its coverage also started to include free-electron laser research (Doniach, 1996 ▸) including instrumentation, theory, computing and scientific applications in areas such as biology, nanoscience and materials science. Just in the last year, authors from 35 different countries published in the journal, the top five countries represented being the USA, Japan, Germany, China and France. Throughout the past 26 plus years *JSR* has been an up-to-date information resource for scientists and engineers in the field of synchrotron radiation.

Now, the *JSR* Main Editors, with the International Union of Crystallography (IUCr) Journals Editor-in-Chief and the IUCr Editorial Office, supported by the IUCr Executive Committee, believe that switching to open access will benefit research in this area by disseminating it more easily and rapidly to the global synchrotron and free-electron laser science communities. The clear goal of this initiative is to induce the smoothest and most research-oriented transformation possible of *JSR* from a behind-paywall subscription-based publishing model to an open-access-based publishing model. All of us increasingly work under conditions in which open access supports researchers in every aspect of their workflow. New detailed instructions on general policies for submission, possible open-access discounts, and other guidelines and templates are discussed at https://journals.iucr.org/s/services/openaccess.html. However, we wish to emphasize that all open-access articles will undergo initial editor assessment and the same rigorous peer review process as at present. The management of the peer review process will continue to focus on the high standards and rapid publication expected for IUCr journals.

At this time, we wish to express our enormous appreciation of *JSR*’s supporting (facility) institutions. We also hope that more supporting institutions will join with *JSR* as we move forward from here. *JSR* supporting institutions are entitled to a certain number of open-access article processing charge (APC) vouchers per year for papers reporting work carried out at their facilities. Alternatively, if the contact author’s home institution is included in a transformative or read-and-publish arrangement with the IUCr’s publication partner, Wiley, those authors will be able to publish open-access research or review articles in *JSR* with no direct (APC) charge. Currently, such arrangements exist in Austria, Finland, Germany, Hungary, Ireland, Italy, Liechtenstein, the Netherlands, Norway, Spain, Sweden, Switzerland and the UK. Contact authors with connections to IUCr (Associates, members of national affiliates, World Directory of Crystallography, *etc*.) will receive modest APC discounts. Meanwhile, discounts (50%) for authors from lower middle income countries and waivers (100%) from low income countries will be issued. However, please note that a major difference from present arrangements is that all submitting authors will need to apply for such discounts and waivers at the time of submission and payments will be handled via Wiley authors services (for more details see https://journals.iucr.org/s/services/openaccess.html).

As the transition to open access proceeds, the *JSR* Editorial Board welcomes feedback from the *JSR* research community, especially from authors, on the new open-access procedures, and is keen to know what is working well and what needs some adjustment.

The change to open access is made with every faith in the future. The Editors fully believe that the publishing of scientific research with global open access providing worldwide visibility without barriers demonstrably leads to more downloads, citations and more impact for authors. The increased citation of open-access articles published in *JSR* since 2018 is shown graphically in the figure ▸.

The *JSR* Editors embrace both the idea and concept of making research freely available to all researchers, and are committed to coordinate and establish best principles to facilitate a smooth transition from subscription to open access. The Editors wish all the best to all *JSR* authors for the future and look forward to seeing your forthcoming high-quality open-access research submissions to *JSR*.

## Figures and Tables

**Figure 1:**
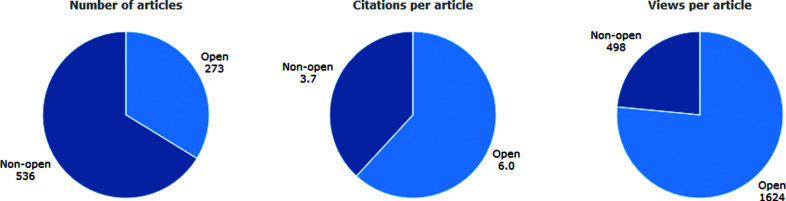
Graphic showing the increased citation of open-access articles published in *JSR* since 2018.
